# 2,6-Dichloro-*N*-phenyl­benzamide

**DOI:** 10.1107/S1600536811019222

**Published:** 2011-06-18

**Authors:** Hui Liu, Ning Xu, Bo Yang, Wei Wang, Ping-fang Han

**Affiliations:** a College of Life Science and Pharmaceutical Engineering, Nanjing University of Technology, Xinmofan Road No. 5 Nanjing, Nanjing 210009, People’s Republic of China; b College of Environment, Nanjing University of Technology, Xinmofan Road No. 5 Nanjing, Nanjing 210009, People’s Republic of China

## Abstract

There are two independent mol­ecules in the asymmetric unit of the title compound, C_13_H_9_Cl_2_NO, in which the dihedral angles between the phenyl and dichloro­phenyl rings have significantly different values [48.5 (3) and 65.1 (3)°]. In the crystal, the mol­ecules are linked *via* inter­molecular N—H⋯O hydrogen bonds into chains running parallel to the *c* axis.

## Related literature

For the synthesis, see: Houlihan *et al.* (1981 ▶). For standard bond lengths, see: Allen *et al.* (1987 ▶). For related structures, see: Cockroft *et al.* (2007 ▶).
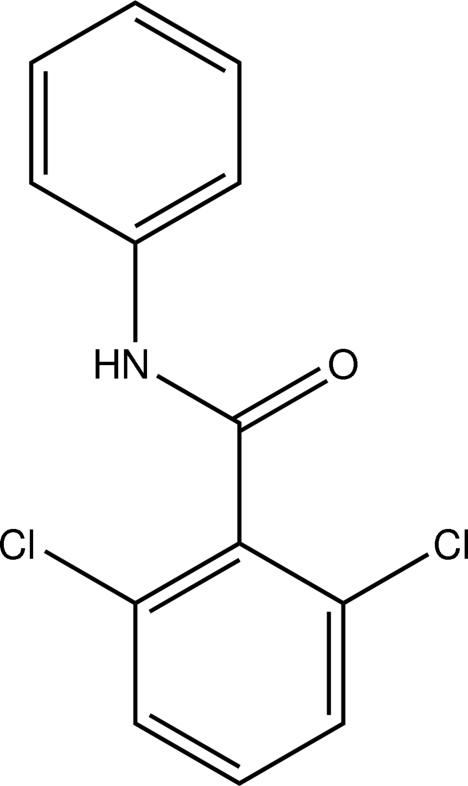


## Experimental

### 

#### Crystal data

C_13_H_9_Cl_2_NO
*M* *_r_* = 266.11
Monoclinic, 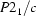

*a* = 12.378 (3) Å
*b* = 11.657 (2) Å
*c* = 17.525 (4) Å
β = 91.43 (3)°
*V* = 2527.9 (9) Å^3^
*Z* = 8
Mo *K*α radiation
μ = 0.50 mm^−1^
*T* = 293 K
0.30 × 0.10 × 0.10 mm

#### Data collection

Enraf–Nonius CAD-4 diffractometer
Absorption correction: ψ scan (North *et al.*, 1968 ▶) *T*_min_ = 0.866, *T*_max_ = 0.952
4807 measured reflections
4586 independent reflections
2154 reflections with *I* > 2σ(*I*)
*R*_int_ = 0.029
3 standard reflections every 200 reflections  intensity decay: 1%

#### Refinement

*R*[*F* ^2^ > 2σ(*F* ^2^)] = 0.067
*wR*(*F* ^2^) = 0.168
*S* = 1.00
4586 reflections
307 parameters
H-atom parameters constrained
Δρ_max_ = 0.24 e Å^−3^
Δρ_min_ = −0.36 e Å^−3^

### 

Data collection: *CAD-4 Software* (Enraf–Nonius, 1989 ▶); cell refinement: *CAD-4 Software*; data reduction: *XCAD4* (Harms & Wocadlo, 1995 ▶); program(s) used to solve structure: *SHELXS97* (Sheldrick, 2008 ▶); program(s) used to refine structure: *SHELXL97* (Sheldrick, 2008 ▶); molecular graphics: *PLATON* (Spek, 2009 ▶); software used to prepare material for publication: *SHELXL97*.

## Supplementary Material

Crystal structure: contains datablock(s) global, I. DOI: 10.1107/S1600536811019222/pv2414sup1.cif

Structure factors: contains datablock(s) I. DOI: 10.1107/S1600536811019222/pv2414Isup2.hkl

Supplementary material file. DOI: 10.1107/S1600536811019222/pv2414Isup3.cml

Additional supplementary materials:  crystallographic information; 3D view; checkCIF report

## Figures and Tables

**Table 1:** Hydrogen-bond geometry (Å, °)

*D*—H⋯*A*	*D*—H	H⋯*A*	*D*⋯*A*	*D*—H⋯*A*
N1—H1*A*⋯O2^i^	0.86	2.09	2.927 (5)	163
N2—H2*B*⋯O1^ii^	0.86	1.99	2.830 (5)	165

Symmetry codes: (i) 
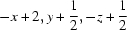
; (ii) 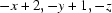.

**Fig. 1.**
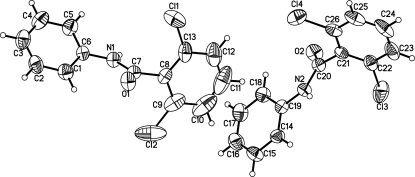
The molecular structure of the title compound showing the atom-numbering scheme and displacement ellipsoids at the 30% probability level.

**Fig. 2.**
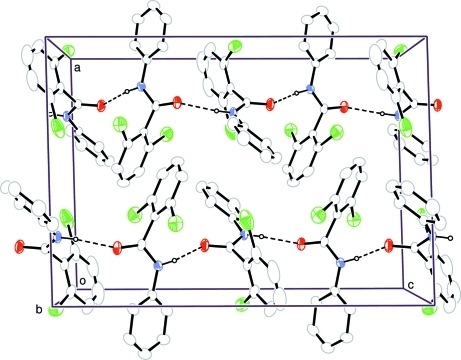
A packing diagram of the title compound. The intermolecular hydrogen bonds are shown as dashed lines; H-atoms not involved in H-bonding have been excluded for clarity.

## supplementary crystallographic information

### Comment

In the title compound (Fig. 1), the bond lengths and angles are within normal ranges (Allen *et al.*, 1987). There are two independent molecules in an asymmetric unit. The dihedral angle between phenyl (C1–C6) and dichlorophenyl (C8–C13) rings in one molecule is 48.5 (3)° compared to 65.1 (3)° in the other molecule. In the crystal structure, intermolecular N—H···O hydrogen bonds (Table 1) link the molecules into chains running parallel to the *c*-axis (Fig. 2). The structure is further stabilized by intramolecular interactions of the type C—H···O (Table 1).

### Experimental

The title compound was prepared by following a reported procedure (Houlihan *et al.*, 1981). A mixture of aniline (2.8 g, 0.03 mol), 2,6-dichlorobenzoyl chloride (6.3 g, 0.03 mol), and 6 ml triethylamine in 50 ml anhydrous tetrahydrofuran was stirred and refluxed for 8 h and then allowed to stand at room temperature. The resulting solid was filtered off and washed with water (2× 30 ml), dried over sodium sulfate (yield: 7.2 g, 90%). The title compound was purified by crystallizing from ethanol yielding crystals suitable for X-ray diffraction analysis.

### Refinement

H atoms were positioned geometrically, with C—H = 0.93 and N—H = 0.86 Å and constrained to ride on their parent atoms, with *U*_iso_(H) = 1.2*U*_eq_(C/N).

### Crystal data

**Table:**

C_13_H_9_Cl_2_NO	*F*(000) = 1088
*M**_r_* = 266.11	*D*_x_ = 1.398 Mg m^−^^3^
Monoclinic, *P*2_1_/*c*	Mo *K*α radiation, λ = 0.71073 Å
Hall symbol: -P 2ybc	Cell parameters from 25 reflections
*a* = 12.378 (3) Å	θ = 9–13°
*b* = 11.657 (2) Å	µ = 0.50 mm^−^^1^
*c* = 17.525 (4) Å	*T* = 293 K
β = 91.43 (3)°	Block, colourless
*V* = 2527.9 (9) Å^3^	0.30 × 0.10 × 0.10 mm
*Z* = 8	

### Data collection

**Table:**

Enraf–Nonius CAD-4 diffractometer	2154 reflections with *I* > 2σ(*I*)
Radiation source: fine-focus sealed tube	*R*_int_ = 0.029
graphite	θ_max_ = 25.3°, θ_min_ = 1.7°
ω/2θ scans	*h* = 0→14
Absorption correction: ψ scan (North *et al.*, 1968)	*k* = 0→14
*T*_min_ = 0.866, *T*_max_ = 0.952	*l* = −21→21
4807 measured reflections	3 standard reflections every 200 reflections
4586 independent reflections	intensity decay: 1%

### Refinement

**Table:**

Refinement on *F*^2^	Primary atom site location: structure-invariant direct methods
Least-squares matrix: full	Secondary atom site location: difference Fourier map
*R*[*F*^2^ > 2σ(*F*^2^)] = 0.067	Hydrogen site location: inferred from neighbouring sites
*wR*(*F*^2^) = 0.168	H-atom parameters constrained
*S* = 1.00	*w* = 1/[σ^2^(*F*_o_^2^) + (0.070*P*)^2^] where *P* = (*F*_o_^2^ + 2*F*_c_^2^)/3
4586 reflections	(Δ/σ)_max_ < 0.001
307 parameters	Δρ_max_ = 0.24 e Å^−^^3^
0 restraints	Δρ_min_ = −0.36 e Å^−^^3^

### Special details

**Table:**

Geometry. All e.s.d.'s (except the e.s.d. in the dihedral angle between two l.s. planes) are estimated using the full covariance matrix. The cell e.s.d.'s are taken into account individually in the estimation of e.s.d.'s in distances, angles and torsion angles; correlations between e.s.d.'s in cell parameters are only used when they are defined by crystal symmetry. An approximate (isotropic) treatment of cell e.s.d.'s is used for estimating e.s.d.'s involving l.s. planes.
Refinement. Refinement of *F*^2^ against ALL reflections. The weighted *R*-factor *wR* and goodness of fit *S* are based on *F*^2^, conventional *R*-factors *R* are based on *F*, with *F* set to zero for negative *F*^2^. The threshold expression of *F*^2^ > σ(*F*^2^) is used only for calculating *R*-factors(gt) *etc*. and is not relevant to the choice of reflections for refinement. *R*-factors based on *F*^2^ are statistically about twice as large as those based on *F*, and *R*- factors based on ALL data will be even larger.

### Fractional atomic coordinates and isotropic or equivalent isotropic displacement parameters (Å^2^)

**Table:**

	*x*	*y*	*z*	*U*_iso_*/*U*_eq_	
Cl1	0.97924 (12)	0.86429 (16)	−0.00070 (10)	0.1283 (6)	
N1	1.2544 (2)	0.8856 (3)	0.00933 (17)	0.0602 (9)	
H1A	1.2464	0.8846	0.0579	0.072*	
O1	1.2151 (2)	0.7888 (3)	−0.10032 (14)	0.0815 (9)	
C1	1.3839 (4)	0.9621 (4)	−0.0806 (3)	0.0815 (13)	
H1B	1.3899	0.8905	−0.1035	0.098*	
Cl2	1.31603 (18)	0.57910 (17)	0.01462 (10)	0.1680 (9)	
C2	1.4429 (4)	1.0533 (5)	−0.1066 (3)	0.0895 (15)	
H2A	1.4888	1.0421	−0.1471	0.107*	
C3	1.4363 (4)	1.1573 (5)	−0.0756 (3)	0.0950 (16)	
H3A	1.4769	1.2179	−0.0942	0.114*	
C4	1.3676 (5)	1.1736 (4)	−0.0149 (3)	0.1019 (17)	
H4A	1.3620	1.2457	0.0075	0.122*	
C5	1.3076 (4)	1.0833 (4)	0.0123 (3)	0.0827 (13)	
H5A	1.2615	1.0945	0.0528	0.099*	
C6	1.3161 (3)	0.9770 (4)	−0.0205 (2)	0.0566 (10)	
C7	1.2075 (3)	0.8004 (3)	−0.0308 (2)	0.0564 (10)	
C8	1.1439 (4)	0.7168 (4)	0.0145 (2)	0.0638 (11)	
C9	1.1858 (5)	0.6147 (5)	0.0379 (3)	0.1027 (19)	
C10	1.1226 (9)	0.5356 (7)	0.0811 (4)	0.147 (3)	
H10A	1.1506	0.4658	0.0981	0.177*	
C11	1.0196 (9)	0.5681 (8)	0.0959 (5)	0.164 (5)	
H11A	0.9770	0.5185	0.1238	0.197*	
C12	0.9770 (6)	0.6660 (7)	0.0728 (3)	0.129 (2)	
H12A	0.9061	0.6841	0.0843	0.155*	
C13	1.0376 (5)	0.7408 (4)	0.0320 (2)	0.0881 (15)	
Cl3	0.66623 (13)	0.16121 (13)	0.29111 (9)	0.1217 (6)	
Cl4	0.69136 (12)	0.58215 (11)	0.17119 (8)	0.1060 (5)	
O2	0.7863 (2)	0.4326 (3)	0.32969 (15)	0.0806 (9)	
N2	0.8572 (2)	0.3470 (3)	0.22519 (16)	0.0549 (8)	
H2B	0.8389	0.3164	0.1821	0.066*	
C14	1.0337 (3)	0.2715 (4)	0.2072 (2)	0.0687 (11)	
H14A	1.0021	0.2178	0.1743	0.082*	
C15	1.1440 (4)	0.2708 (4)	0.2186 (3)	0.0863 (14)	
H15A	1.1866	0.2176	0.1935	0.104*	
C16	1.1907 (4)	0.3504 (5)	0.2678 (3)	0.0919 (15)	
H16A	1.2651	0.3510	0.2762	0.110*	
C17	1.1277 (4)	0.4273 (5)	0.3037 (3)	0.0875 (15)	
H17A	1.1596	0.4807	0.3366	0.105*	
C18	1.0159 (4)	0.4280 (4)	0.2922 (2)	0.0740 (12)	
H18A	0.9735	0.4811	0.3174	0.089*	
C19	0.9687 (3)	0.3490 (3)	0.2431 (2)	0.0562 (10)	
C20	0.7758 (3)	0.3869 (3)	0.2669 (2)	0.0579 (10)	
C21	0.6667 (3)	0.3711 (4)	0.2297 (2)	0.0549 (10)	
C22	0.6084 (4)	0.2723 (4)	0.2402 (2)	0.0726 (12)	
C23	0.5063 (5)	0.2590 (5)	0.2082 (3)	0.0966 (17)	
H23A	0.4684	0.1911	0.2154	0.116*	
C24	0.4610 (4)	0.3457 (7)	0.1659 (3)	0.109 (2)	
H24A	0.3915	0.3377	0.1452	0.131*	
C25	0.5177 (4)	0.4442 (6)	0.1540 (3)	0.0988 (18)	
H25A	0.4874	0.5027	0.1244	0.119*	
C26	0.6198 (4)	0.4571 (4)	0.1857 (2)	0.0722 (12)	

### Atomic displacement parameters (Å^2^)

**Table:**

	*U*^11^	*U*^22^	*U*^33^	*U*^12^	*U*^13^	*U*^23^
Cl1	0.1012 (11)	0.1715 (16)	0.1128 (12)	0.0422 (11)	0.0185 (9)	0.0025 (11)
N1	0.071 (2)	0.075 (2)	0.0347 (16)	−0.0089 (19)	0.0004 (15)	−0.0037 (16)
O1	0.096 (2)	0.110 (2)	0.0395 (16)	−0.0249 (19)	0.0091 (14)	−0.0131 (16)
C1	0.086 (3)	0.092 (4)	0.067 (3)	−0.003 (3)	0.011 (3)	−0.003 (3)
Cl2	0.227 (2)	0.1771 (17)	0.0973 (12)	0.1220 (17)	−0.0434 (12)	−0.0265 (11)
C2	0.082 (3)	0.112 (4)	0.076 (3)	−0.012 (3)	0.016 (3)	0.022 (3)
C3	0.103 (4)	0.099 (4)	0.082 (4)	−0.024 (4)	−0.009 (3)	0.028 (3)
C4	0.134 (5)	0.073 (4)	0.098 (4)	−0.017 (4)	−0.005 (4)	−0.008 (3)
C5	0.100 (4)	0.079 (3)	0.069 (3)	−0.011 (3)	0.005 (3)	−0.005 (3)
C6	0.058 (2)	0.069 (3)	0.043 (2)	−0.006 (2)	−0.0025 (19)	0.006 (2)
C7	0.065 (3)	0.064 (3)	0.041 (2)	0.000 (2)	−0.0014 (19)	−0.004 (2)
C8	0.094 (3)	0.058 (3)	0.039 (2)	−0.012 (3)	−0.001 (2)	0.000 (2)
C9	0.177 (6)	0.077 (4)	0.053 (3)	0.011 (4)	−0.020 (3)	0.000 (3)
C10	0.277 (11)	0.083 (5)	0.080 (5)	−0.002 (7)	−0.042 (6)	0.012 (4)
C11	0.269 (13)	0.133 (8)	0.091 (5)	−0.096 (9)	−0.009 (7)	0.028 (5)
C12	0.142 (6)	0.162 (7)	0.084 (4)	−0.058 (6)	0.031 (4)	−0.007 (4)
C13	0.108 (4)	0.109 (4)	0.048 (3)	−0.043 (3)	0.014 (3)	−0.002 (3)
Cl3	0.1448 (13)	0.1094 (11)	0.1111 (11)	−0.0318 (10)	0.0069 (9)	0.0454 (9)
Cl4	0.1334 (12)	0.0765 (9)	0.1080 (10)	0.0172 (8)	−0.0024 (9)	0.0099 (8)
O2	0.092 (2)	0.109 (2)	0.0412 (15)	−0.0032 (19)	0.0053 (14)	−0.0202 (16)
N2	0.056 (2)	0.067 (2)	0.0414 (17)	0.0034 (17)	−0.0032 (15)	−0.0082 (15)
C14	0.067 (3)	0.063 (3)	0.076 (3)	0.003 (2)	−0.005 (2)	−0.009 (2)
C15	0.061 (3)	0.103 (4)	0.095 (4)	0.014 (3)	0.000 (3)	−0.006 (3)
C16	0.067 (3)	0.113 (4)	0.095 (4)	0.000 (3)	−0.010 (3)	0.005 (3)
C17	0.079 (4)	0.103 (4)	0.080 (3)	−0.016 (3)	−0.023 (3)	−0.007 (3)
C18	0.072 (3)	0.081 (3)	0.069 (3)	−0.002 (3)	−0.010 (2)	−0.011 (2)
C19	0.063 (3)	0.060 (3)	0.045 (2)	−0.003 (2)	−0.0066 (19)	0.0000 (19)
C20	0.068 (3)	0.066 (3)	0.039 (2)	0.001 (2)	0.008 (2)	0.000 (2)
C21	0.055 (2)	0.068 (3)	0.042 (2)	0.007 (2)	0.0118 (18)	−0.006 (2)
C22	0.068 (3)	0.091 (4)	0.059 (3)	−0.005 (3)	0.015 (2)	−0.006 (3)
C23	0.074 (4)	0.130 (5)	0.088 (4)	−0.020 (4)	0.032 (3)	−0.027 (4)
C24	0.049 (3)	0.182 (7)	0.097 (4)	0.011 (4)	0.000 (3)	−0.047 (5)
C25	0.069 (4)	0.143 (6)	0.083 (4)	0.039 (4)	−0.008 (3)	−0.021 (4)
C26	0.066 (3)	0.088 (3)	0.062 (3)	0.021 (3)	0.006 (2)	−0.011 (3)

### Geometric parameters (Å, °)

**Table:**

Cl1—C13	1.704 (6)	Cl3—C22	1.719 (5)
N1—C7	1.341 (4)	Cl4—C26	1.728 (5)
N1—C6	1.418 (5)	O2—C20	1.226 (4)
N1—H1A	0.8600	N2—C20	1.343 (4)
O1—C7	1.232 (4)	N2—C19	1.409 (4)
C1—C2	1.374 (6)	N2—H2B	0.8600
C1—C6	1.374 (5)	C14—C19	1.373 (5)
C1—H1B	0.9300	C14—C15	1.375 (5)
Cl2—C9	1.723 (7)	C14—H14A	0.9300
C2—C3	1.331 (7)	C15—C16	1.384 (6)
C2—H2A	0.9300	C15—H15A	0.9300
C3—C4	1.393 (7)	C16—C17	1.354 (6)
C3—H3A	0.9300	C16—H16A	0.9300
C4—C5	1.380 (6)	C17—C18	1.393 (6)
C4—H4A	0.9300	C17—H17A	0.9300
C5—C6	1.371 (6)	C18—C19	1.380 (5)
C5—H5A	0.9300	C18—H18A	0.9300
C7—C8	1.494 (5)	C20—C21	1.496 (5)
C8—C9	1.358 (6)	C21—C22	1.374 (5)
C8—C13	1.387 (6)	C21—C26	1.383 (5)
C9—C10	1.437 (10)	C22—C23	1.378 (6)
C10—C11	1.361 (11)	C23—C24	1.367 (7)
C10—H10A	0.9300	C23—H23A	0.9300
C11—C12	1.317 (10)	C24—C25	1.365 (8)
C11—H11A	0.9300	C24—H24A	0.9300
C12—C13	1.365 (7)	C25—C26	1.375 (6)
C12—H12A	0.9300	C25—H25A	0.9300
			
C7—N1—C6	126.4 (3)	C20—N2—C19	128.2 (3)
C7—N1—H1A	116.8	C20—N2—H2B	115.9
C6—N1—H1A	116.8	C19—N2—H2B	115.9
C2—C1—C6	119.8 (5)	C19—C14—C15	121.9 (4)
C2—C1—H1B	120.1	C19—C14—H14A	119.0
C6—C1—H1B	120.1	C15—C14—H14A	119.0
C3—C2—C1	122.1 (5)	C14—C15—C16	118.9 (5)
C3—C2—H2A	119.0	C14—C15—H15A	120.5
C1—C2—H2A	119.0	C16—C15—H15A	120.5
C2—C3—C4	118.7 (5)	C17—C16—C15	119.9 (5)
C2—C3—H3A	120.6	C17—C16—H16A	120.1
C4—C3—H3A	120.6	C15—C16—H16A	120.1
C5—C4—C3	120.3 (5)	C16—C17—C18	121.2 (5)
C5—C4—H4A	119.9	C16—C17—H17A	119.4
C3—C4—H4A	119.9	C18—C17—H17A	119.4
C6—C5—C4	119.8 (5)	C19—C18—C17	119.3 (4)
C6—C5—H5A	120.1	C19—C18—H18A	120.4
C4—C5—H5A	120.1	C17—C18—H18A	120.4
C5—C6—C1	119.4 (4)	C14—C19—C18	118.8 (4)
C5—C6—N1	118.5 (4)	C14—C19—N2	118.0 (3)
C1—C6—N1	122.1 (4)	C18—C19—N2	123.2 (4)
O1—C7—N1	123.9 (4)	O2—C20—N2	125.1 (4)
O1—C7—C8	120.6 (4)	O2—C20—C21	121.3 (4)
N1—C7—C8	115.6 (3)	N2—C20—C21	113.7 (3)
C9—C8—C13	117.9 (5)	C22—C21—C26	117.9 (4)
C9—C8—C7	122.0 (5)	C22—C21—C20	121.1 (4)
C13—C8—C7	120.1 (4)	C26—C21—C20	121.0 (4)
C8—C9—C10	120.8 (7)	C21—C22—C23	121.3 (5)
C8—C9—Cl2	119.5 (5)	C21—C22—Cl3	119.2 (4)
C10—C9—Cl2	119.7 (6)	C23—C22—Cl3	119.4 (5)
C11—C10—C9	116.5 (8)	C24—C23—C22	119.8 (5)
C11—C10—H10A	121.7	C24—C23—H23A	120.1
C9—C10—H10A	121.7	C22—C23—H23A	120.1
C12—C11—C10	123.6 (10)	C25—C24—C23	119.9 (5)
C12—C11—H11A	118.2	C25—C24—H24A	120.0
C10—C11—H11A	118.2	C23—C24—H24A	120.0
C11—C12—C13	119.6 (8)	C24—C25—C26	120.1 (6)
C11—C12—H12A	120.2	C24—C25—H25A	119.9
C13—C12—H12A	120.2	C26—C25—H25A	119.9
C12—C13—C8	121.6 (6)	C25—C26—C21	121.0 (5)
C12—C13—Cl1	118.8 (5)	C25—C26—Cl4	120.1 (4)
C8—C13—Cl1	119.5 (4)	C21—C26—Cl4	118.9 (4)
			
C6—C1—C2—C3	−0.3 (8)	C19—C14—C15—C16	0.2 (7)
C1—C2—C3—C4	0.1 (8)	C14—C15—C16—C17	−0.1 (8)
C2—C3—C4—C5	0.0 (8)	C15—C16—C17—C18	0.2 (8)
C3—C4—C5—C6	0.2 (8)	C16—C17—C18—C19	−0.3 (7)
C4—C5—C6—C1	−0.5 (7)	C15—C14—C19—C18	−0.4 (6)
C4—C5—C6—N1	179.2 (4)	C15—C14—C19—N2	176.9 (4)
C2—C1—C6—C5	0.5 (7)	C17—C18—C19—C14	0.4 (6)
C2—C1—C6—N1	−179.2 (4)	C17—C18—C19—N2	−176.8 (4)
C7—N1—C6—C5	143.6 (4)	C20—N2—C19—C14	158.0 (4)
C7—N1—C6—C1	−36.8 (6)	C20—N2—C19—C18	−24.8 (6)
C6—N1—C7—O1	2.4 (6)	C19—N2—C20—O2	−0.4 (6)
C6—N1—C7—C8	−178.1 (4)	C19—N2—C20—C21	179.5 (4)
O1—C7—C8—C9	81.7 (5)	O2—C20—C21—C22	−91.6 (5)
N1—C7—C8—C9	−97.9 (5)	N2—C20—C21—C22	88.5 (4)
O1—C7—C8—C13	−96.4 (5)	O2—C20—C21—C26	86.2 (5)
N1—C7—C8—C13	84.1 (5)	N2—C20—C21—C26	−93.7 (4)
C13—C8—C9—C10	−1.6 (7)	C26—C21—C22—C23	−0.2 (6)
C7—C8—C9—C10	−179.7 (5)	C20—C21—C22—C23	177.7 (4)
C13—C8—C9—Cl2	177.8 (3)	C26—C21—C22—Cl3	177.3 (3)
C7—C8—C9—Cl2	−0.3 (6)	C20—C21—C22—Cl3	−4.9 (5)
C8—C9—C10—C11	0.9 (10)	C21—C22—C23—C24	−0.8 (7)
Cl2—C9—C10—C11	−178.5 (6)	Cl3—C22—C23—C24	−178.2 (4)
C9—C10—C11—C12	0.0 (13)	C22—C23—C24—C25	1.4 (8)
C10—C11—C12—C13	−0.2 (13)	C23—C24—C25—C26	−1.2 (8)
C11—C12—C13—C8	−0.6 (9)	C24—C25—C26—C21	0.2 (7)
C11—C12—C13—Cl1	177.3 (6)	C24—C25—C26—Cl4	−179.6 (4)
C9—C8—C13—C12	1.5 (7)	C22—C21—C26—C25	0.5 (6)
C7—C8—C13—C12	179.6 (4)	C20—C21—C26—C25	−177.4 (4)
C9—C8—C13—Cl1	−176.4 (3)	C22—C21—C26—Cl4	−179.8 (3)
C7—C8—C13—Cl1	1.7 (5)	C20—C21—C26—Cl4	2.4 (5)

### Hydrogen-bond geometry (Å, °)

**Table:**

*D*—H···*A*	*D*—H	H···*A*	*D*···*A*	*D*—H···*A*
N1—H1A···O2^i^	0.86	2.09	2.927 (5)	163
N2—H2B···O1^ii^	0.86	1.99	2.830 (5)	165
C1—H1B···O1	0.93	2.46	2.920 (7)	110
C18—H18A···O2	0.93	2.41	2.937 (6)	116

Symmetry codes: (i) −*x*+2, *y*+1/2, −*z*+1/2; (ii) −*x*+2, −*y*+1, −*z*.

## References

[bb1] Allen, F. H., Kennard, O., Watson, D. G., Brammer, L., Orpen, A. G. & Taylor, R. (1987). *J. Chem. Soc. Perkin Trans. 2*, pp. S1–19.

[bb2] Cockroft, S. L., Perkins, J., Zontas, C., Adams, H., Spey, S. E., Low, C. M. R., Vinter, J. G., Lawson, K. R., Urch, C. J. & Hunter, C. A. (2007). *Org. Biomol. Chem.* **5**, 1062–1080. 10.1039/b617576g 17377660

[bb3] Enraf–Nonius (1989). *CAD-4 Software* Enraf–Nonius, Delft, The Netherlands.

[bb4] Harms, K. & Wocadlo, S. (1995). *XCAD4* University of Marburg, Germany.

[bb5] Houlihan, W. J., Uike, Y. & Parrino, V. A. (1981). *J. Org. Chem* **46**, 4515–17.

[bb6] North, A. C. T., Phillips, D. C. & Mathews, F. S. (1968). *Acta Cryst.* A**24**, 351–359.

[bb7] Sheldrick, G. M. (2008). *Acta Cryst.* A**64**, 112–122. 10.1107/S0108767307043930 18156677

[bb8] Spek, A. L. (2009). *Acta Cryst.* D**65**, 148–155. 10.1107/S090744490804362X PMC2631630 19171970

